# A protocol for applying health equity-informed implementation science models and frameworks to adapt a sleep intervention for adolescents at risk for suicidal thoughts and behaviors

**DOI:** 10.3389/fpubh.2022.971754

**Published:** 2022-10-12

**Authors:** Ariel A. Williamson, Adriane M. Soehner, Rhonda C. Boyd, Daniel J. Buysse, Allison G. Harvey, Charles R. Jonassaint, Peter L. Franzen, Tina R. Goldstein

**Affiliations:** ^1^Department of Child and Adolescent Psychiatry and Behavioral Sciences, Children's Hospital of Philadelphia, Philadelphia, PA, United States; ^2^Department of Psychiatry, Perelman School of Medicine, University of Pennsylvania, Philadelphia, PA, United States; ^3^Department of Psychiatry, University of Pittsburgh School of Medicine, Pittsburgh, PA, United States; ^4^Department of Psychology, University of California, Berkeley, Berkeley, CA, United States; ^5^Department of Medicine, University of Pittsburgh School of Medicine, Pittsburgh, PA, United States

**Keywords:** adolescent, adaptation, circadian, health equity, intervention, sleep, suicide, implementation science

## Abstract

**Background:**

Effective and equitable strategies to prevent youth suicidal thoughts and behaviors (STB) are an urgent public health priority. Adolescent sleep disturbances are robustly linked to STB but are rarely addressed in preventive interventions or among Black and/or Hispanic/Latinx youth for whom STB risk is increasing disproportionately. This paper describes an application of health equity-informed implementation science models and frameworks to adapt and evaluate the evidence-based Transdiagnostic Sleep and Circadian (TSC) intervention for primary care implementation with adolescents of minoritized backgrounds with depression and STB risk.

**Methods:**

This multiphase study protocol uses the Assessment, Decision, Adaptation, Production, Topical Experts-Integration, Training, Testing (ADAPT-ITT) model to adapt and evaluate TSC for primary care implementation with adolescents who are depressed, at risk for STB, and of primarily Black and/or Hispanic/Latinx backgrounds. We integrate the Consolidated Framework for Implementation Research (CFIR) in an initial qualitative inquiry of adolescent, caregiver, and clinician perceptions of TSC. Subsequent ADAPT-ITT phases include systematically and iteratively testing adaptations based on the qualitative inquiry, with ongoing key informant input, and then evaluating the adapted TSC for feasibility, acceptability, and efficacy in a pilot randomized trial.

**Anticipated results:**

Based on youth depression and sleep health disparities research, we expect that TSC adaptations will be needed to enhance intervention content for adolescents with depression, STB risk, and primarily Black and/or Hispanic/Latinx backgrounds. We also anticipate adaptations will be needed to align TSC delivery methods with primary care implementation.

**Conclusions:**

Adapting evidence-based interventions with end-users and contexts in mind can help ensure that intervention strategies and delivery methods are acceptable to, and feasible with, health disparate populations. Although TSC has shown effectiveness for adolescents with sleep disturbances, we expect that additional multiphase research is necessary to optimize TSC for primary care delivery with Black and/or Hispanic/Latinx adolescents with depression and STB risk.

## Introduction

Youth suicide is a significant public health concern, ranking as the second leading cause of death for young people worldwide (1). In the United States, suicide attempts and deaths have increased more rapidly among African American, Caribbean American, and other Black American (hereafter referred to as “Black”) youth compared to any other racial or ethnic group (2, 3). Disproportionate increases in suicide risk are also apparent in Hispanic/Latinx youth (hereafter, ‘Latinx’), underscoring the need for preventive efforts that are culturally tailored to address these disparities (4). However, few effective interventions exist for adolescent suicidal thoughts and behaviors (STB) (5), and those that are available have been tested with youth of primarily non-Hispanic/Latinx White (hereafter, ‘White’) backgrounds (6). This research gap raises questions about whether such treatments are similarly effective among youth of minoritized backgrounds, or whether culturally responsive adaptations would enhance effectiveness. These open questions and observed racial and ethnic disparities reflect an urgent need for effective and equitable STB prevention in adolescence.

### Sleep as an optimal target of adolescent STB prevention

To effectively prevent adolescent STB, interventions must focus on risk factors that are acute, proximal, and modifiable (7). Sleep disturbances are among the few risk factors that meet these criteria, but are rarely addressed in preventive interventions for youth STB (8, 9). A range of subjective sleep and circadian problems (e.g., insomnia symptoms, poor perceived sleep quality, sleeping much of the day, daytime sleepiness) and objective indicators of poor sleep health (e.g., short sleep duration, high variability, late bedtimes) (10) are robustly associated with the continuum of STB (11, 12), from suicidal ideation (13) to death by suicide (14). In addition to these temporal linkages with STB, sleep disturbances are implicated in the onset and maintenance of depressive symptoms in adolescence (15, 16), one of the strongest risk factors for youth STB (17). Moreover, sleep disturbances are modifiable, with a growing body of research supporting the efficacy of cognitive and behavioral approaches in treating youth sleep problems as well as comorbid mood concerns (8, 18). Findings from adult research demonstrate the potential for sleep treatment to improve STB. Two randomized controlled trials (RCTs) have shown that cognitive-behavioral (19) or pharmacological treatment (20) of insomnia yields post-treatment reductions in STB among adults, supporting the value of addressing sleep disturbances to prevent STB.

### Adolescent sleep health disparities

Racial and ethnic sleep health disparities are well-documented in adolescence (21). Sleep-related risk factors for increased STB, such as a short sleep duration, poor sleep quality, and variable sleep timing, are more prevalent among Black and/or Latinx youth compared to White youth (21–24). Both social and environmental factors contribute to sleep health disparities. Black youth are more likely than their White peers to live in lower socioeconomic status (SES) homes and neighborhoods (25, 26), which can contribute to poor sleep *via* environmental factors including high levels of light, noise, household crowding, and community violence (27–30). In addition, among Black and/or Latinx youth, exposure to racism and discrimination at multiple levels (i.e., systemic/institutional; personally mediated; internalized) (31–34) can contribute to sleep difficulties, including long sleep onset latency and poor sleep quality (35, 36). For example, in a study of Black, Latinx, and Asian American youth, experiences of discrimination were associated with same-day sleep disturbances (37). It is also possible that stressors related to racism and discrimination exacerbate the adverse impacts of sleep-disrupting environmental factors (30). For instance, in one study community violence concerns were linked to short and poor quality sleep in Black but not White adolescents, who most likely do not experience daily discrimination (38).

### The need for a culturally tailored sleep intervention for youth with STB risk

**Figure 1:**
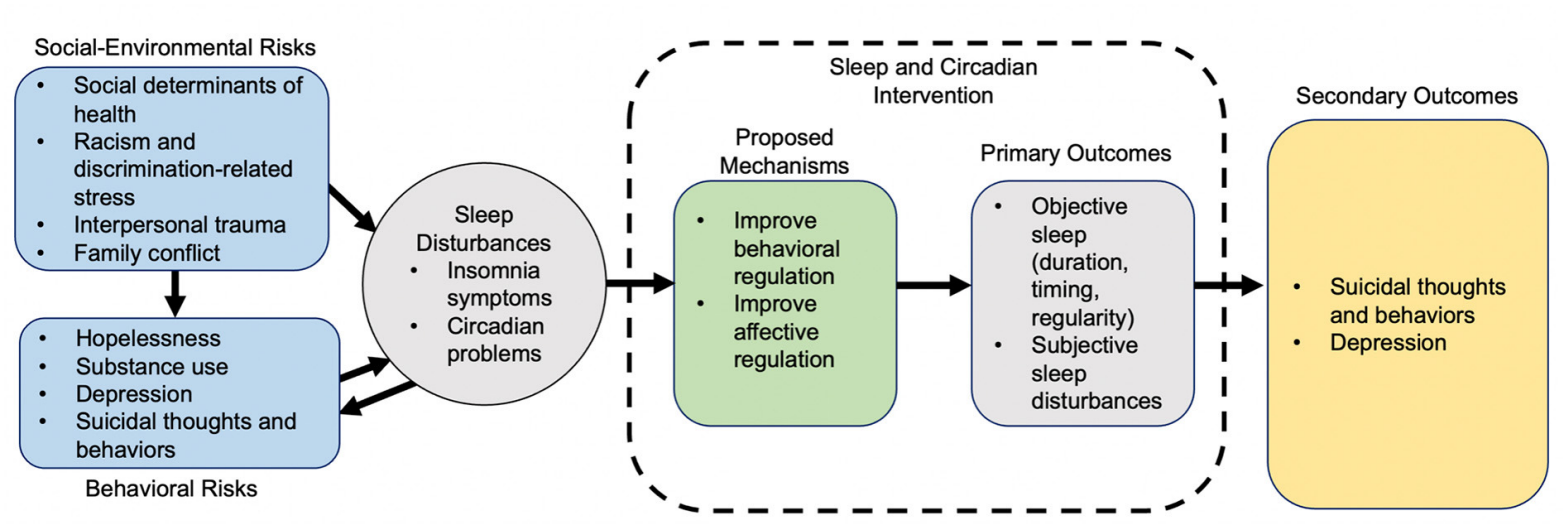
Conceptual model of adolescent risk factors, sleep disturbances, depression, STB risk, and sleep intervention mechanisms.

Experiencing more sleep problems compared to their White counterparts may put Black and/or Latinx youth at increasingly higher risk for depression and STB (39). Figure 1 presents a proposed conceptual model in which social-environmental risks, including social determinants of health, racism, and discrimination, and well-established behavioral risks factors (e.g., prior STB, hopelessness, depression) (3, 39) collectively contribute to sleep and circadian disturbances and, ultimately, STB risk *via* proximal affective and behavioral dysregulation. Accordingly, treating sleep disturbances could improve affective and behavioral regulation, in turn reducing depression and risk for STB (8, 9). A sleep-focused intervention to decrease STB risk may be especially well-suited for Black and/or Latinx youth with depression and sleep disturbance, given stigmatization of mental health treatment (40, 41).

To date, however, very few sleep treatments have been tested with Black and/or Latinx youth (42). The few studies testing adolescent sleep interventions with Black and/or Latinx youth have shown lower acceptability (43) as well as diminished treatment response (44) in these groups compared to White youth. These poorer outcomes could be due to limited attention to salient socio-cultural and environmental factors (45), including the adverse impacts of racism and discrimination on sleep in minoritized youth (35–37). To ensure acceptability and effectiveness, a sleep intervention for Black and/or Latinx adolescents with depression and at risk for STB must be tailored to address these socio-cultural and environmental factors and disparities. In addition, most youth with psychiatric disorders present with comorbid conditions and a range of sleep disturbances (46, 47), such as insomnia symptoms and the (frequently co-occurring) circadian rhythm disruptions that are highly prevalent in adolescence (48). Thus, for a sleep intervention to be effective with a diverse population, it must also be transdiagnostic with regard to both sleep and psychiatric concerns.

### The proposed research

The Transdiagnostic Sleep and Circadian intervention (TSC, also referred to as TranS-C) is one of the only evidence-based treatments designed to treat a range of sleep and circadian difficulties among individuals with psychiatric comorbidities (46, 47). Grounded in a dimensional model of sleep health (49), TSC builds on principles of basic sleep and circadian science, evidence-based CBT strategies, and a motivational interviewing framework (46, 47), in which the patient is viewed as the expert in behavior change to enhance personal responsibility (50). TSC is modularized to enable flexible delivery and tailoring to each patient's specific sleep and circadian difficulties (51). In a community-based RCT, TSC was effective in treating sleep disturbances among adults who had comorbid sleep and psychiatric concerns (52). In this study, Black adults in particular experienced a strong treatment benefit (53). Another RCT conducted with predominantly White adolescents with delayed circadian rhythms showed that TSC produced durable improvements in sleep and circadian disturbances, even at 12-month follow-up (54–56).

TSC has not yet been tested among youth who are depressed and at risk for STB, with primarily Black and/or Latinx adolescents, or in primary care, where behavioral health services may be more accessible for minoritized youth (57, 58). Adaptations to intervention content (i.e., treatment strategies) and delivery methods (i.e., implementation strategies) are likely needed to maximize TSC acceptability, effectiveness, uptake, and scaling. Baumann and Cabassa (59, 60) recommend embedding implementation science with a health equity lens to adapt evidence-based interventions with end-users and contexts in mind, to ensure that intervention content and delivery methods are acceptable to and feasible with health disparate populations. This approach can also help to avoid perpetuating the well-documented gaps in the translation and uptake of evidence-based interventions in clinical practice settings (61, 62).

**Figure 2:**
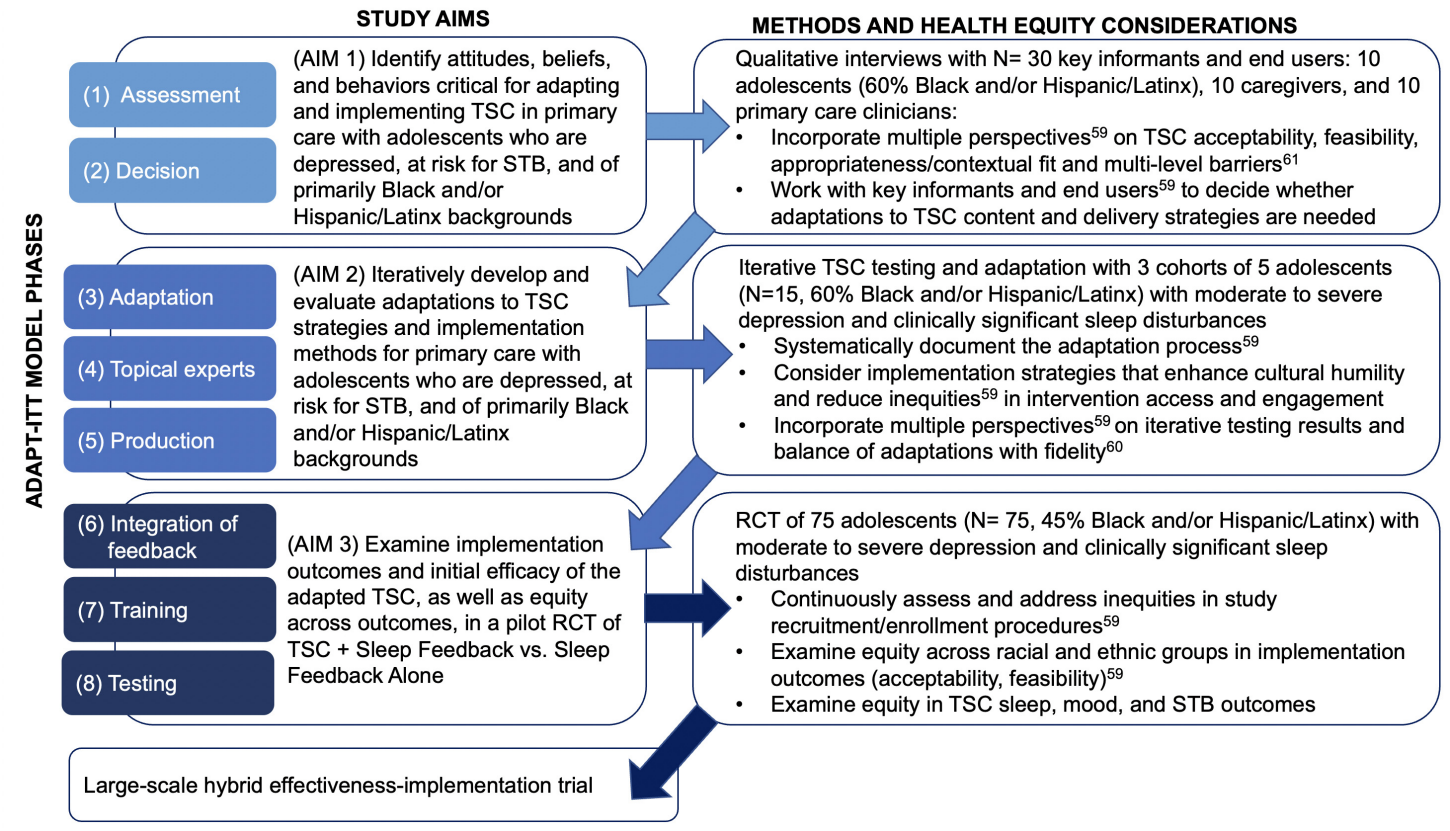
ADAPT-ITT phases applied to multiphase protocol aims, methods, and health equity considerations.

Following these recommendations, the purpose of this paper is to describe a protocol for applying health-equity informed implementation science frameworks to systematically adapt and evaluate with adolescents who are depressed, at risk for STB, and of primarily Black and/or Latinx backgrounds. Specifically, we use the Assessment, Decision, Adaptation, Production, Topical Experts-Integration, Training, Testing (ADAPT-ITT) model (63) to guide our multiphase, iterative adaptation and evaluation of TSC for this new clinical population and implementation context (Figure 2). We also integrate the Consolidated Framework for Implementation Research (CFIR) (61) to ensure assessment of, and adaptations for, contextual barriers and facilitations of implementation, such as clinician practices and organizational factors. In the following sections, we present preliminary data showing the acceptability and feasibility of TSC with a small sample of adolescents who are depressed and at risk for STB. We then describe the three sequential aims and protocol for the planned multiphase TSC adaptation and evaluation, which includes initial qualitative interviews with key informants, iterative TSC adaptation, and a pilot RCT to evaluate the adapted TSC intervention.

## Initial pilot findings

### Methods

We first conducted an open trial using a convenience sample to examine preliminary feasibility and acceptability of TSC with adolescents who were experiencing depression and suicidal ideation. Standard TSC includes 8–12 sessions consisting of modules shown in Table 1. *Cross-cutting*, process-focused modules (case formulation, education, motivational enhancement, and goal setting) are included in each session. These cross-cutting modules are supplemented by *core modules* that apply to most patients (establishing regular sleep-wake times, learning a wind-down/wake-up routine, improving daytime functioning, addressing unhelpful sleep-related beliefs, and maintenance of behavior change), and *optional modules* for additional intervention personalization (improving sleep efficiency, reducing time in bed, dealing with delayed or advanced phase, and reducing sleep-related worry/vigilance) (47). Given prominent circadian timing changes in adolescence and late circadian preference in >75% of adolescents with depression, we enhanced TSC prior to implementation to further stabilize circadian rhythmicity (71). To this end, we integrated daily light therapy (target of 30 min in the morning delivered with Re-Timer glasses) to increase morning bright light exposure, and blue light-blocking glasses (up to 2 h before bedtime) to reduce evening light, particularly that from electronics devices. We also integrated sleep feedback through graphs constructed from sleep diaries and wrist actigraphy and used these data and participants' subjective sleep complaints to support ongoing case formulation, goal setting, and the selection of core and optional TSC modules. Adaptations to TSC for youth with depression and suicidal ideation were iteratively made, and qualitative and quantitative feedback from youth were incorporated to yield a personalized intervention.

**Table 1:** TSC intervention content, potential adaptations, and scientific rationale for adaptations.

**Content**	**Potential adaptations**	**Scientific rationale for adaptations**
**Cross-cutting modules**		
Case formulation	Integrate American Psychiatric Association's (APA) Cultural Formulation Interview (CFI) content (64, 65).	• Helps ensure enhancements to cultural and contextual fit are assessed and included at treatment initiation (66).
Education		
Behavior change		
Motivation		
**Core modules**		
Establishing regular sleep-wake times	Adjust recommendations and increase problem-solving for: • Work, school, and childcare schedules (67). • Characteristics of sleep environment in lower-SES homes and/or neighborhoods (27, 28).	• Flexibility to address youth/family barriers to consistent sleep schedules and routines (66, 68). • Environment plays important role in sleep of youth in lower-SES homes and/or neighborhoods (27, 28).
Learning a wind-down/ wake-up routine		
Improving daytime functioning	Coping strategies for contextual factors, including: • Racism and discrimination for minoritized youth (34, 37). • Limited resources/opportunities for youth of lower-SES backgrounds (69, 70).	• Social and environmental factors contribute to disparities in adolescent sleep health (30, 37, 45). • Problem-solving/coping strategies may be beneficial (13).
Correcting unhelpful sleep-related beliefs	• Assess alignment with family sleep beliefs/preferences • Integrate APA CFI content (64, 65).	• Promotes tailoring of intervention strategies to youth/family culture (66).
Maintenance of behavior change	• Discuss adolescent-specific transitions (e.g., summer to school term, middle school to high school) • Anticipate mood fluctuations	• School start times significantly impact adolescent sleep (48). • Youth at risk for STB may experience mood fluctuations that disrupt sleep gains (65).
**Optional modules**		
Improving sleep efficiency	Adjust recommendations and increase problem-solving for social and environmental sleep disruptors, including light, noise, neighborhood, and family factors	• Adolescent sleep associated with social (safety, crime) and environmental (light, noise) neighborhood factors (27). • Family environment can reduce youth sleep efficiency (29).
Reducing time in bed	Problem-solve limited opportunities for time out of bed based on sleep space and family context	• Family factors can impact youths' ability to remain out of bed (e.g., room-/bed-sharing) (29).
Dealing with delayed or advanced phase	Provide evening blue blocker glasses and morning re-timer goggles+ (71).	• Neighborhood-level variation in light exposure (27). • Sleep environment factors (e.g., room-/bed-sharing) (28, 29).
Reducing sleep-related worry/vigilance	Apply cognitive strategies (coping, restructuring) to address pre-sleep worry/vigilance related to racism, discrimination, and neighborhood safety concerns	• Racism, discrimination, neighborhood violence linked with poor sleep quality and bedtime hyperarousal (36, 38, 39). • Targeting these may benefit sleep onset and quality

+ indicates adaptation made during open pilot of TSC.

Participant inclusion criteria were age 13–18 years, able to understand and converse in English, receiving care at a specialty clinic for youth at high risk for STB, with current moderate sleep disturbance [Pittsburgh Sleep Quality Index (72) global score >8], current suicidal ideation (per clinician report or self-report), depression, and a parent/guardian willing to consent for research. Adolescents were excluded if they had a bipolar disorder diagnosis, or were taking any photo-sensitizing medications (e.g., neuroleptics and antiarrhythmic drugs), as the bright light administered in in our adapted version of TSC is contraindicated with these medications. Participating parents/guardians provided informed consent and adolescents provided assent; youth who were age 18 or turned 18 during the study provided informed consent. Participants wore a wrist actigraph (CentrePoint Insight Watch, Actigraph Corp, Pensacola FL), completed daily sleep diaries (items described elsewhere), and attended TSC sessions every 1–3 weeks with a master's level study clinician, who delivered the program as an adjunct to youths' behavioral healthcare in the specialty clinic. The study was approved by the Institutional Review Board.

### Results

Fifteen adolescents (M age = 16.1 years, SD = 1.6; 94% White and 6% Black; all non-Latinx) completed an average of 5.1 (SD = 2.6) TSC sessions (range = 1–10 sessions). Within-person average completion rates of morning and evening diaries occurred on 68 and 67% of days, respectively. Youth self-reported adherence on the daily diary to the ReTimer and blue-blocker intervention strategies was 56 and 59% of study days (among the completed diary days), respectively. For the five teens who completed acceptability ratings post-intervention, the overall mean satisfaction with the quality of TSC was 6.2 (range: 6–7) on a scale from one “very dissatisfied” to seven “very satisfied.” The mean rating of whether youth would recommend TSC to a friend who had sleep difficulties was 6.4 (range: 6–7) on a scale from one “strongly not recommended” to seven “strongly recommended,” while the mean reported likelihood of using the information and strategies learned about sleep in the future was 6.0 (range: 5–7) on a scale from 1 “not at all” to seven “very much.” Free text feedback included, “I enjoyed the bright light goggles and tracking my activity and its interactions with my sleep;” “I enjoyed the sleep therapy sessions. I found them to be helpful and beneficial to my sleep and routine;” “It helped with checking on my status of sleep and track how little and much I was sleeping;” and the morning bright light goggles “helped me get out of bed and ‘jumpstart’ my day.” All participants who completed TSC reported the length was appropriate (all rated as a four, on a scale from 1 “much too short,” four “appropriate,” to seven “much too long”).

**Figure 3:**
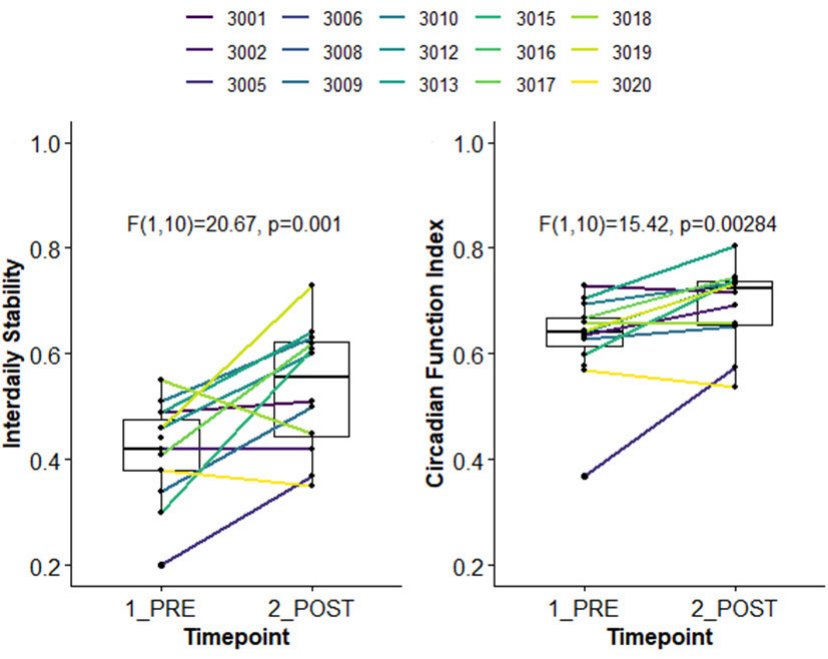
Pre-to-post improvement in 24-h rest activity rhythms in open pilot study.

Using the first and last week of available actigraphy data, we examined change in 24-h rest activity rhythms (RARs) (73, 74), indexed by non-parametric outcomes (nparACT R package) (75). Interdaily Stability (IS) captures the degree of stability in the 24-h activity rhythm from day-to-day, varying from zero (unstable, noise) to one (stable, same activity pattern every day); here, the 24 h profile was estimated using 30-min time bins. The Circadian Function Index (CFI) (76) is a composite measure of circadian robustness, calculated as the average of three nparACT outcomes: IS, Relative Amplitude (ratio of highest 10 h of activity to lowest 5 h of activity), and inverted/normalized Intradaily Variability (within-day rhythm fragmentation). CFI ranges between zero (absence of circadian rhythmicity) and one (a robust circadian rhythm). As shown in Figure 3, compared to the baseline week, youth had significantly higher stability in 24-h activity patterns (i.e., in IS and CFI), suggesting improvement in 24-h RARs.

### Implications for multiphase protocol

The open pilot establishes preliminary feasibility and acceptability of TSC with adolescents who are depressed and experiencing suicidal ideation. Results also suggest that TSC can improve 24-h RARs. However, adolescents in this sample were recruited through a specialty care clinic and were already engaged in behavioral healthcare, potentially increasing the likelihood of TSC session attendance. To tailor TSC for primary care delivery, adaptations to the number and duration of sessions may be needed, given the brief nature of primary care-based behavioral healthcare (57). Pilot results were also limited to mostly White, non-Latinx adolescents. Research on youth suicide prevention (5, 6), sleep health disparities (21, 45), and the impact of racism and/or discrimination (36, 37, 39) supports the likelihood that adaptations to TSC content will be needed to enhance its cultural relevance for Black and/or Latinx youth.

## Multiphase TSC adaptation and evaluation protocol

### Design

Figure 2 shows the application of the 8-phase ADAPT-ITT model (63) to the three sequential aims of the planned study, as well as considerations to enhance health equity (59). ADAPT-ITT prioritizes key informant and end-user feedback in iterative, sequential adaptations of evidence-based interventions. First developed for use in HIV prevention and intervention research, ADAPT-ITT has been applied to adaptations of many other evidence-based interventions, including a treatment for adolescent substance use (77) and a universal suicide prevention program in pediatric primary care (78). The use of ADAPT-ITT is relevant to the proposed adaptation of TSC to ensure that intervention content and delivery strategies are acceptable, feasible, and culturally responsive in a new population (i.e., adolescents who are depressed, at risk for STB, and of primarily Black and/or Latinx backgrounds) and a new context (i.e., primary care) (63).

In line with the first two ADAPT-ITT phases, Assessment and Decision (63), Aim 1 identifies attitudes, beliefs, and behaviors critical for adapting and implementing TSC in the new population and new context described above. We will qualitatively solicit the perspectives of key informants, end-users, and patients/clients, including primary care and behavioral health clinicians, adolescent patients, and their caregivers. We will use CFIR (61) to guide the development of interview questions about contextual/organizational barriers and facilitators to intervention content and delivery strategies. These methods align with prior research on adapting sleep intervention strategies for primary care implementation with minoritized young children and families (67). In our analyses and interpretation of results, we will incorporate the perspectives of the multiple key informant groups (59) to better understand the feasibility, appropriateness, contextual fit, and potential multi-level barriers (61) to TSC implementation. These results will directly inform decisions about which adaptations to TSC intervention content and delivery strategies would be necessary to maximize intervention acceptability, feasibility, and efficacy in the target population and clinical context.

Table 1 shows potential adaptations based on prior research, including our sleep intervention implementation and adaptation research (54, 55, 66, 67, 71). Potential adaptations include integrating questions from the American Psychiatric Association's Cultural Formulation Interview (64, 65) to ask about prior experiences with and preferences for enhancing cultural fit from the beginning of treatment (66). Training for TSC interventionists will likely require adaptation to more explicitly focus on enhancing interventionists' cultural humility (79) and awareness of how clinician implicit and explicit racial and ethnic biases impact the clinical encounter (80). Interventionist training may also benefit from content-related adaptations to address the impacts of racism and discrimination on sleep (35–37). More specifically, the optional module on reducing sleep-related worry and/or hypervigilance could be adapted so that cognitive coping techniques are applied to adolescents' experiences of and stress related to racism and discrimination (Table 1). Based on prior research (27–29, 66), we also anticipate that adaptations to TSC may be required for youth of lower-SES backgrounds. For instance, problem-solving can focus on potentially modifiable social and environmental sleep disruptors in the adolescent sleep environment, including light, noise, and lack of privacy for youth who room-/bed-share (27–29).

ADAPT-ITT phases three, four, and five consist of a “theater test,” in which the intervention is implemented with the target population (Administration), with additional intervention adaptation (Production), and continued input on any adaptations from key informants (Topical Experts) (63). These phases will be accomplished through Aim 2, which iteratively develops and evaluates adaptations to TSC strategies and implementation methods with primarily Black and/or Latinx adolescents recruited from primary care. The initial adaptations suggested in Aim 1 will be iteratively tested in cohorts of adolescents in Aim 2, with additional adaptations drawn from ongoing intervention participants, topical experts, and clinical advisory board feedback (Figure 2). As in Aim 1 and in line with health equity recommendations (59), we will incorporate this feedback in interpreting the Aim 2 results, as well as in balancing adaptations made with fidelity (60) to the TSC intervention. Importantly, throughout this iterative testing we will systematically document the nature and extent of any adaptations made (59).

We will then complete ADAPT-ITT phases six (Integration of feedback), seven (Training), and eight (Testing) through Aim 3, which examines implementation outcomes and initial efficacy of the adapted TSC, as well as equity across outcomes, in a pilot RCT. Activities for this aim (Figure 2) include implementing a finalized version of the adapted TSC based on the results of prior aims, ensuring adequate and adapted training as needed for TSC interventionists, and examining any observed disparities by racial and ethnic group with regard to study procedures (e.g., recruitment, enrollment) and clinical outcomes (59).

### Setting and recruitment

All study aims will be conducted in pediatric primary care practices affiliated with two large academic medical centers in PA. The affiliated pediatrics practices in western PA serve over 70% of youth in the region and include 32 practices across 54 office sites in nine western PA counties. This group of practices serves over 266,000 privately and publicly insured patients, aged birth to 21 years. The second affiliated primary care network in eastern PA is the largest provider of primary care services in the region, with 31 practices across five counties. Clinicians in the network serve over 249,000 private and publicly insured patients, aged birth to 21 years. For each study aim, we will obtain caregiver/guardian consent, adolescent assent and/or consent for adolescents aged 18 years.

To ensure that the participant groups (adolescents, caregivers, and primary care clinicians) across study aims are reflective of the target clinical population and implementation context, we will recruit participants as part of routine well child visits. Prior to visits, potentially eligible patients based on electronic health record (EHR) screening will receive an email jointly signed by a primary care practice champion at the family's care site and research staff providing information about the research. During well child visits, the Patient Health Questionnaire (PHQ-9-M) (81) depression screener is administered as part of standard practice and integrated into the EHR. For all study phases, we will implement EHR-based clinician-directed alerts if patient scores meet criteria for potential enrollment based on their depressive symptoms. If the patient and family are interested in the research, we will conduct either in-person or remote informed consent procedures to maximize flexibility for participants. Throughout study phases, we will monitor the recruitment of Black and/or Hispanic/Latinx adolescents and caregivers and meet regularly with primary care clinical teams to solicit ongoing feedback on recruitment procedures and adjust these methods as needed to recruit a diverse sample. The protocol for this study was approved by the Institutional Review Boards of the affiliated academic medical centers. The following sections detail additional methods by aim.

### Aim 1: Initial qualitative inquiry

#### Aim 1 participants

Aim 1 participants will include 10 adolescents, 10 caregivers of adolescents, and 10 primary care clinicians, including physicians and behavioral health providers, who are working at the affiliated primary care sites. We aim to recruit a sample with at least 50% of adolescent and caregiver participants self-identifying as Black and at least 10% self-identifying as Latinx. Adolescent inclusion criteria are as follows: age 12–18 years; able to understand and converse in English; and evidence of moderate-severe depression (PHQ-9-M >11) (81) and clinically significant sleep disturbance (PHQ-9-M sleep item 3 [“trouble falling or staying asleep”] >2, sleep trouble >50% of days in past 2 weeks). Participants with a life-threatening medical condition requiring immediate treatment or intellectual or developmental disability precluding comprehension of study procedures will be excluded, as will adolescent participants with diagnoses of obstructive sleep apnea, restless legs syndrome, bipolar disorder, a current manic or psychotic episode (per participant or caregiver report or medical record review).

#### Aim 1 measures

Interview guides will be developed with input from key informant groups, including adolescents, caregivers, providers, and health systems leaders to ensure the most relevant information is gathered. The dimensional CFIR framework (61) will guide interview questions on multi-level barriers and facilitators to TSC content and delivery strategies. More specifically, in the CFIR domain of intervention characteristics, we will solicit perspectives on the relative advantage of implementing TSC in primary care vs. other outpatient settings, the extent to which the content would require adaptation to meet adolescents' needs, and the ways in which the intervention is packaged and delivered to youth. Related to the CFIR outer setting domain, we will ask interviewees about the needs and resources of adolescents seen in the primary care sites. Inner setting questions will inquire about the norms and values of the primary care setting and the climate for implementation (61). This includes questions about the likelihood of future intervention implementation, dissemination, and sustainment (82) in primary care after the research concludes. In the CFIR domain of individual characteristics we will solicit perspectives about adolescents' sleep-related knowledge, beliefs, and attitudes, as well as their views of the TSC intervention content and planned delivery methods. We will embed a health equity perspective (59) and assess youth experiences of personally-mediated and systemic/institutional racism and discrimination, the impacts of these experiences on sleep, and their perceptions about addressing the sleep impacts of these experiences through TSC (31–34).

#### Aim 1 analytic approach

The sample size for this aim was based on guidelines for thematic saturation in qualitative research (83). We will focus our analysis on the *a priori* attributes of interest, specifically CFIR domains, TSC barriers, and potential adaptations (84, 85). We will initially analyze interviews using Rapid Qualitative Analysis (86), to facilitate the rapid analysis and iterative adaptation of intervention content and delivery strategies. During data collection and analysis, we will assess for thematic saturation and for a diversity of perspectives given the small number of participants proposed and the focus of this research on racially and ethnically minoritized youth. If necessary, we will increase our sample size to maximize the inclusion of a wide range of perspectives within and across key informant groups. To incorporate qualitative data from the different interview groups (adolescents; caregivers; clinicians) we will follow NIH guidelines (87) and mixed methods approaches (88) to stratify the themes that emerge according to informant groups. This will require interview transcription, the iterative development of a codebook, and the coding of qualitative data in a specialized software program.

### Aim 2: Iterative TSC adaptation

#### Aim 2 participants

We will recruit 15 adolescents (at least 50% self-identifying as Black; 10% self-identifying as Latinx) in three cohorts of five adolescents each to participate in iteratively adapting and testing TSC. Aim 2 inclusion/exclusion criteria are identical to those in Aim 1.

#### Aim 2 measures

The primary Aim 2 outcomes pertain to intervention acceptability and feasibility. We will also pilot the sleep data collection methods and strategies to increase morning bright light and decrease evening light in anticipation of the Aim 3 randomized trial. Adolescents will wear actigraphs, Re-Timer glasses in the morning, blue-blocker glasses in the evening, complete a daily sleep diary, and provide ratings of their perceived sleep disturbances at pre and post intervention.

*Intervention acceptability* will be assessed at post-intervention using the adolescent self-reported Acceptability of Intervention Measure (AIM) (89). We will also conduct semi-structured qualitative interviews to identify participants' perspectives about intervention acceptability, barriers, and recommendations for additional adaptations to content or delivery strategies.

*Intervention feasibility* will be indexed by multiple outcomes (90), including the number of TSC sessions attended and rates of intervention attrition, to index intervention engagement. Intervention fidelity will be measured *via* the coding of a randomly selected 10% of video recorded TSC sessions. Sessions will be coded using the Cognitive Therapy Scale (CTS) (91) and a TSC session checklist (92), both used in prior TSC research.

*Actigraphy* is a widely used method of assessing sleep and circadian disruptions longitudinally in an individual's natural environment. Actigraphy is well-validated validated against polysomnography, the gold standard measure of overnight sleep (93). Consistent with guidelines, adolescents will continuously wear an actigraph on their non-dominant wrist, unless bathing or swimming, and complete a corresponding sleep diary for actigraphy scoring purposes. Sleep diary ratings will include time in and out of bed, sleep onset latency, night awakenings, and sleep quality (94). Actigraphy data will be scored using the Cole-Kripke algorithm in ActiLife software, which are validated against polysomnography and other actigraphs in young adults (95) and adolescents (96).

*Self-reported sleep disturbances* will be measured using the well-validated pediatric Patient-Reported Outcomes Measurement Information Systems (PROMIS) Sleep Disturbance and Sleep-Related Impairment Scales (97), which respectively measure perceived sleep difficulties and the impacts of sleep on daily functioning.

#### Aim 2 intervention procedures

TSC study clinicians will review the intervention manual and attend an initial 1-day training with the study investigators, who will conduct weekly supervision. The TSC developers (AGH and DJB) will consult on implementation as needed. Clinicians will interface with adolescents' other treatment providers and/or their caregivers in line with preferences identified in Aim 1. Therapist fidelity ratings (described above) will be monitored, with ratings < 80% prompting re-training with study investigators.

Study clinicians will implement core and optional modules weekly over 6–8 weeks *via* a HIPAA-compliant, secure telehealth platform. TSC sessions will supplement other mental health treatment that participants may be receiving. The selection and individualization of TSC models will be guided by the intake assessment, case conceptualization, adolescents' ongoing reports of sleep disturbances at sessions, and their actigraphy and daily diary data.

#### Aim 2 analytic approach

Our prior adaptation research (63, 66) and guidelines for thematic saturation (83) informed the Aim 2 sample size. As in Aim 1, we will monitor participant TSC ratings and qualitative feedback and increase our sample size if we do not reach thematic saturation. Aggregate mean AIM scores will be reviewed following each cohort of five participants. Scores for the final cohort will quantify overall acceptability, with high end-user acceptability identified as a mean AIM >80%.

### Aim 3: Pilot RCT of adapted TSC

#### Aim 3 participants and randomization

We will recruit 75 adolescents (at least 35% self-identifying as Black; 10% self-identifying as Latinx) to participate in the RCT comparing the adapted TSC intervention plus Sleep Feedback (TSC + Sleep Feedback, described below) to a Sleep Feedback Only condition. Study inclusion/exclusion criteria are identical to those in Aim 1. Adolescents will be eligible to enroll in the study while engaged in other behavioral/mental healthcare and/or taking any sleep medications, which we will track.

Adolescents will be randomized using 2:1 allocation (2 TSC + Sleep Feedback: 1 Sleep Feedback Only) using a modification of Efron's biased coin toss procedure (98). We selected unequal allocation to maximize critical information about the intervention (e.g., adverse events). Random assignment will balance groups on age (middle vs. high school, since school start times typically shift earlier and social pressures further shorten sleep duration in high school), suicide risk (ideation/attempt history), and racial and ethnic background.

#### Aim 3 measures

Primary Aim 3 outcomes are related to TSC implementation, and include intervention feasibility, acceptability, appropriateness, and fidelity. Secondary Aim 3 outcomes are adolescent sleep disturbances, depressive symptoms, STB, and affective and behavioral regulation; the last of these are hypothesized intervention mechanisms (Figure 1). Sleep will be objectively assessed using actigraphy, which adolescents will wear throughout the TSC intervention period, with an accompanying daily diary to assess self-reported sleep, mood, and stressors (described below). All other secondary outcomes will be collected at baseline (pre-intervention) and at months 1, 3, 6, and 12. The type, frequency, and/or dose of behavioral, sleep, psychiatric treatment and/or medications will also be measured throughout the study using the Child and Adolescent Services Assessment (CASA) (99) throughout the study. Specific primary and secondary measures are as follows:

*Intervention acceptability* will be measured using the adolescent self-reported AIM (89) instrument, described in Aim 2. To further assess acceptability, adolescents will also complete the Client Satisfaction Questionnaire (CSQ) (100), adapted for the current study, as well as a semi-structured qualitative interview, with questions about TSC as described for the Aim 2 post-intervention interviews.

*Intervention feasibility* will be assessed through multiple methods (90), including *via* engagement (TSC sessions attended and attrition) and intervention fidelity assessments described for Aim 2. Adolescents will also complete the Intervention Appropriateness Measure (IAM) and the Feasibility of Intervention Measure (FIM) (89) to further assess perceived fit and feasibility of the intervention for addressing their sleep disturbances, respectively.

*Actigraphy* will be used to evaluate behavioral sleep and circadian characteristics, with the same procedures for implementation and scoring as in Aim 2. Outcomes for actigraphy-derived sleep disturbances are duration, regularity, and timing. Consistent with Study 1, a sleep diary will be used to complement actigraphy metrics for scoring purposes. The sleep diary will include time in and out of bed, sleep onset latency, night awakenings, and sleep quality (94). The sleep-specific diary questions will be sent *via* SMS text messages to participants each morning. See below for additional diary items administered in the evening.

*Daily mood and stressors* will be measured *via* adolescent self-reported daily diary items implemented during the TSC intervention period. These items will be deployed using links to a web-based form sent *via* SMS texts or emails, as in the sleep diary implementation. These items will be assessed in the evening and will include ratings of adolescents' mood; experiences of racism, discrimination, and victimization (101); and affective and behavioral regulation (impulsivity and reactivity to the day's most positive and negative event as in our prior work) (102).

*Weekly self-reported suicidal ideation and behavior* will also be rated *via* SMS using items modeled after the Columbia—Suicide Severity Rating Scale (C-SSRS) (103, 104).

*Weekly affective and behavioral regulation*, which are hypothesized intervention mechanisms, will also be measured through adolescent self-report using the Childhood Affective Lability Scale (CALS) (105) to assess affective regulation and the short UPPS-P Impulsive Behavior Scale (IBS) (106) for behavioral regulation.

*Self-reported sleep disturbances* will be measured at baseline and follow-up assessments using the PROMIS Sleep Disturbance and Sleep-Related Impairment Scales (97), as in Aim 2.

*Self-reported depressive symptoms and STB* will also be measured at baseline and follow-up assessments using the adolescent PHQ-9-M (81) and the C-SSRS, (103, 104) respectively.

#### Aim 3 intervention procedures

Intervention training and implementation procedures for TSC will be as described for Aim 2. The Sleep Feedback Only condition consists of reports summarizing prospectively gathered actigraphy and diary data. With the mass availability of wearable devices (e.g., Fitbit) and apps, such personalized sleep tracking is now widely accessible. However, despite increasing users' awareness of sleep habits, this approach yields minimal change in sleep behavior (107, 108). Thus, the Sleep Feedback Only comparator group controls for common receipt of information related to sleep behaviors while enabling us to focus on TSC adaptations to optimize ultimate implementation. These Sleep Feedback reports will be accessible to participants *via* web link sent weekly by SMS. Sleep Feedback reports will also be accessible to TSC clinicians *via* a HIPAA-secured online portal to inform selection and personalization of TSC modules.

#### Aim 3 analytic approach

As Aim 3 is a pilot study, sample size considerations center on the precision of confidence interval (CI) width estimation for implementation and target outcomes. Based on best practices for pilot studies (109, 110), given our intervention sample size (TSC + Sleep Feedback) of 50 and 5% type I error rate, we will be able to estimate 95% CI widths of no more than 0.28 for primary implementation and target outcomes.

Using descriptive statistics, we will compute the proportion (and 95% confidence intervals) of participants with high ratings for TSC feasibility (session attendance >80%, attrition < 20%; FIM >80%), acceptability (AIM >80%) and appropriateness (IAM >80%). We will examine these outcomes overall and according to participant racial and ethnic groups. For implementation outcomes with both quantitative and qualitative data (feasibility, acceptability, and appropriateness), we will use established approaches for analyzing mixed methods data described as in Aim 1. We will compare participants across cells on clinical and socio-demographic baseline characteristics using standard univariate statistics.

Additionally, we will assess whether improvements in sleep (*via* actigraphy-derived duration, regularity, timing; and *via* daily diary and PROMIS measures), depressive symptoms (PHQ-9-M), and risk for STB (C-SSRS), are greater among youth randomized to TSC + Sleep Feedback vs. Sleep Feedback Only conditions using linear mixed models. Exploratory analyses will examine putative intervention mechanisms (affective/behavioral dysregulation; Figure 1) based on daily diary, CALS, and IBS ratings. Study arm, time, and their interaction will be included as primary predictors, with random effects for study subject.

## Discussion

This paper describes a protocol for applying health equity-informed implementation science frameworks and models to adapt and evaluate the evidence-based, modularized TSC intervention in primary care with adolescents who are depressed, at risk for STB, and of primarily Black and/or Latinx backgrounds. This protocol expands upon our recent open pilot of TSC with predominantly White youth experiencing sleep disturbance and suicidal ideation, which demonstrated preliminary intervention feasibility. Minor adaptations to TSC during the pilot included integrating clinician feedback to youth on their sleep from both sleep diaries and actigraphy, and enhancing morning bright light exposure and evening blue light-blocking glasses, based on our prior research (71). Adolescents reported good adherence to these strategies on the daily diary, as well as high acceptability of these strategies, although the sample size for the post-intervention acceptability questionnaire was modest. Youth in the open pilot also showed evidence of improved 24-h rest activity rhythms.

Our planned multiphase protocol will rigorously develop and evaluate further adaptations of TSC for Black and/or Latinx youth who are treated in primary care settings. Developing adolescent behavioral healthcare that is both evidence-based and accessible is an urgent public health priority (111), particularly given the rising global prevalence of youth anxiety and depression over the course of the coronavirus (COVID-19) pandemic (112). Primary care may be a more accessible and less stigmatizing context for initiating behavioral healthcare (40, 41). The American Academy of Pediatrics also recommends routine adolescent depression screening as part of well child care (113), which facilitates early identification of youth at risk for depression and STB. Despite these benefits, substantial challenges remain to integrating behavioral health screening and referrals into the primary care workflow (114).

These challenges necessitate a CFIR-informed, pre-implementation inquiry to identify organizational and other contextual factors that are critical for intervention delivery methods and future sustainment (61, 82). Throughout the multiphase study, we will monitor whether planned implementation and research methods, such as the use of telehealth (115) and our initial focus on English-speaking families (116), inadvertently contribute to disparities in access to treatment for adolescents and their families presenting to primary care. In addition, we will measure participants' engagement in study evaluations (i.e., actigraphy and daily diaries) and the TSC intervention (i.e., session attendance), as these methods may require further adaptation to better align with the brief and less intensive nature of primary care-based service delivery. Our study will add to a growing body of research examining the feasibility and benefits of evidence-based adolescent behavioral health programs adapted for primary care delivery (117).

We anticipate that the results of the multiphase protocol will ensure adaptations to TSC are made to both optimize delivery methods for primary care and to maximize acceptability, feasibility, and effectiveness with adolescents of primarily Black and/or Latinx backgrounds. Although we have outlined potential intervention content adaptations based on relevant research (5, 9, 36, 37), any proposed cultural adaptations based on race and ethnicity are inherently limited. Race and ethnicity are socio-political constructs (118), and no racial or ethnic group is monolithic; considerable heterogeneity exists within racial and ethnic groups and along many other identity dimensions (e.g., gender identity and expression, language, religion, nationality, ability, etc.). Some of these dimensions, such as race and gender identity, may intersect to confer increased marginalization, and this intersectional lens (119) is needed to better personalize and enhance the cultural fit of any behavioral health treatment (120). Findings for the proposed research may have limited generalizability for these reasons, and due to the small proposed sample sizes across aims and the potential that only 10% of adolescent and caregiver participants may identify as Latinx and only 50% of participants may identify as Black for each aim.

Tailoring an intervention for every possible combination of intersectionality is not feasible and could further limit dissemination and uptake (121), particularly in under-resourced community settings where clinicians may not have time or access to needed trainings (47). At the same time, TSC is a modularized treatment that could facilitate attention to intersectionality with personalization (e.g., tailoring strategies to address adolescents' specific cultural and contextual sources of sleep disruption) across a range of sleep and circadian disturbances and comorbid psychiatric conditions (47). The modularized nature of TSC and the planned adaptations in this research could provide a foundation for the integration of suggested clinician and systems-level adaptations in the TSC training activities and treatment manuals, potentially overcoming the need for evaluating multiple adaptations in future research. Our qualitative, pre-implementation inquiry about TSC content and delivery strategies may also result in other adaptations that could enhance the cultural fit of other modules (e.g., integrating culture-specific beliefs around sleep in the module for correcting unhelpful sleep-related thoughts/beliefs). Indeed, we intend to use the results of this research to inform a fully-powered hybrid effectiveness-implementation trial (122) of TSC in primary care, to further establish the evidence base for TSC adaptations and to examine implementation outcomes with integrated behavioral health providers. Throughout this protocol, we may identify additional intervention content and delivery methods that require tailoring for optimal implementation and effectiveness.

Our research plan provides an example of how health equity-informed implementation science models (ADAPT-ITT) and frameworks (CFIR) can be applied to increase the likelihood that evidence-based interventions will be effective for health disparity populations and successfully implemented in accessible intervention contexts (59). Our goal is to ensure adaptations to TSC are systematically documented, rigorously tested, and developed with end-users in mind, so that this intervention can be scaled to equitably and effectively address adolescent STB.

## Data availability statement

The datasets generated and/or analyzed during the current study are not available for use outside of the University of Pittsburgh at this time, due to the nature of the ethics board approvals and possible risk(s) to study participants as well as the confidentiality promised to them. Data may be made available from the corresponding author on reasonable request with permission of study investigators and ethics board approval. Requests to access the datasets should be directed to AS, soehneram2@upmc.edu.

## Ethics statement

The studies involving human participants were reviewed and approved by Institutional Review Board, School of Medicine, University of Pittsburgh, Pittsburgh, PA. Written informed consent to participate in this study was provided by the participants' legal guardian/next of kin.

## Author contributions

Study conceptualization and design: AW, AS, RB, CJ, PF, and TG. Data collection, analysis, and interpretation of results for pilot test: AS, PF, and TG. Draft manuscript preparation: AW, AS, PF, and TG. Critical manuscript review and feedback: RB, CJ, DB, and AH. All authors contributed to the article and approved the submitted version.

## Funding

This study was supported by K23 HD094905 (AW), P50 MH115838 (Brent/Rollman), R01 MH1224907 (TG/PF), R01 MH118312 (PF), K01 MH124828 (AS).

## Conflict of interest

Over the past 3 years, author DB has served as a paid consultant to National Cancer Institute, Pear Therapeutics, Sleep Number, Idorsia, Eisai, and Weight Watchers International. Author DB is an author of the Pittsburgh Sleep Quality Index, Pittsburgh Sleep Quality Index Addendum for PTSD (PSQI-A), Brief Pittsburgh Sleep Quality Index (B-PSQI), Daytime Insomnia Symptoms Scale, Pittsburgh Sleep Diary, Insomnia Symptom Questionnaire, and RU_SATED (copyrights held by University of Pittsburgh). These instruments have been licensed to commercial entities for fees. He is also co-author of the Consensus Sleep Diary (copyright held by Ryerson University), which is licensed to commercial entities for a fee. The remaining authors declare that the research was conducted in the absence of any commercial or financial relationships that could be construed as a potential conflict of interest.

## Publisher's note

All claims expressed in this article are solely those of the authors and do not necessarily represent those of their affiliated organizations, or those of the publisher, the editors and the reviewers. Any product that may be evaluated in this article, or claim that may be made by its manufacturer, is not guaranteed or endorsed by the publisher.
